# Brain size reductions associated with endothelin B receptor mutation, a cause of Hirschsprung’s disease

**DOI:** 10.1186/s12868-021-00646-z

**Published:** 2021-06-19

**Authors:** Ko-Chin Chen, Zan-Min Song, Geoffrey D. Croaker

**Affiliations:** 1 Medical School, Australian National University, Canberra, ACT 2601 Australia; 2 The Canberra Hospital, Yamba Drive, Garran, ACT 2605 Australia

**Keywords:** Neuroanatomy, Endothelin-B mutation, Neural impairment, Spotting-lethal rat, Hirschsprung disease

## Abstract

**Background:**

ET_B_ has been reported to regulate neurogenesis and vasoregulation in foetal development. Its dysfunction was known to cause HSCR, an aganglionic colonic disorder with syndromic forms reported to associate with both small heads and developmental delay. We therefore asked, "is CNS maldevelopment a more general feature of ET_B_ mutation?" To investigate, we reviewed the micro-CT scans of an ET_B_^−/−^ model animal, *sl/sl* rat, and quantitatively evaluated the structural changes of its brain constituents.

**Methods:**

Eleven neonatal rats generated from ET_B_^+/−^ cross breeding were sacrificed. Micro-CT scans were completed following 1.5% iodine-staining protocols. All scans were reviewed for morphological changes. Selected organs were segmented semi-automatically post-NLM filtering: TBr, T-CC, T-CP, OB, Med, Cer, Pit, and S&I Col. Volumetric measurements were made using Drishti rendering software. Rat genotyping was completed following analysis. Statistical comparisons on organ volume, organ growth rate, and organ volume/bodyweight ratios were made between *sl/sl* and the control groups based on autosomal recessive inheritance. One-way ANOVA was also performed to evaluate potential dose-dependent effect.

**Results:**

*sl/sl* rat has 16.32% lower body weight with 3.53% lower growth rate than the control group. Gross intracranial morphology was preserved in *sl/sl* rats. However, significant volumetric reduction of 20.33% was detected in TBr; similar reductions were extended to the measurements of T-CC, T-CP, OB, Med, and Pit. Consistently, lower brain and selected constituent growth rates were detected in *sl/sl* rat, ranging from 6.21% to 11.51% reduction. Lower organ volume/bodyweight ratio was detected in *sl/sl* rats, reflecting disproportional neural changes with respect to body size. No consistent linear relationships exist between ET_B_ copies and intracranial organ size or growth rates.

**Conclusion:**

Although ET_B_^−/−^ mutant has a normal CNS morphology, significant size reductions in brain and constituents were detected. These structural changes likely arise from a combination of factors secondary to dysfunctional ET-1/ET-3/ET_B_ signalling, including global growth impairment from HSCR-induced malnutrition and dysregulations in the neurogenesis, angiogenesis, and cerebral vascular control. These changes have important clinical implications, such as autonomic dysfunction or intellectual delay. Although further human study is warranted, our study suggested comprehensive managements are required for HSCR patients, at least in ET_B_^−/−^ subtype.

**Supplementary Information:**

The online version contains supplementary material available at 10.1186/s12868-021-00646-z.

## Background

Endothelin receptor B (ET_B_) is a widely expressed seven-transmembrane G-protein coupled receptor (GPCR) with multiple regulatory controls on embryological development, vasoregulation, and endothelin metabolism [1–4]. Although ET_B_ mutation has been well documented to cause Hirschsprung’s disease (HSCR), a paediatric intestinal disease affecting 1/5000 live births, we suspect concurrent intracranial changes is also likely due to the impaired neurogenesis [1, 5, 6]. In this study, we evaluated the neuroanatomical changes associated with ET_B_ mutation by studying the changes detected in spotting-lethal (*sl/sl*) rat, a HSCR animal model. We hope these findings can provide additional evidence to support ET_B_’s importance on anatomical development and the notion that HSCR is likely a multi-organ disease, at least in ET_B_^−/−^ subtype.

Although HSCR is best known for its clinical manifestations of intestinal dilatation and toxic megacolon sequelae, multi-organ dysfunctions have also been documented [7, 8]. Indeed, clinical impairments of central nervous system and autonomic system have been exemplified by HSCR-associated syndromes, including Down syndrome, Waardenburg-Shah syndrome (WS-IV), Haddad syndrome, Goldberg-Shprintzen, and Smith-Lemli-Opitz syndrome [5, 9–12]. This is partially attributable to the polygenetic causes of HSCR, including mutations in several common genes: proto-oncogene RET (RET), glial cell line derived neurotrophic factor (GDNF), endothelin B-receptor (ET_B_), endothelin-3 (ET-3), SOX10, endothelin converting enzyme-1 (ECE-1), neurturin (NTN), and Smad interacting protein 1 (SIP1) genes [12, 13]. Among these, RET mutations are the most common, accounting for 50% of familial cases and 15–20% of sporadic cases of HSCR. This is followed by mutations in ET_B_ (5%) and ET-3 genes (< 5%) [13, 14]. Puffenberger et al. (1994), however, demonstrated a W276C missense mutation in ET_B_ gene of an inbred kindred can result 74% and 21% risks for HSCR development in homozygotes and heterozygotes, respectively [15]. This suggested potential dose effect of ET_B_ and the likely underestimation in its true impact on HSCR and embryological development, possibly due to ET_B_’s multi-regulatory functions. This notion is further supported by the increasing identifications of novel ET_B_ mutations and polymorphism in sporadic HSCR patients, which further emphasizes its developmental importance [16, 17].

Endothelin receptors (A and B; abbreviated respectively as ET_A_ and ET_B_) have been reported to involve in the neurogenesis of both central and peripheral nervous systems. Mutations in endothelin receptors and endothelin (1, 2, and 3; abbreviated respectively as ET-1, 2, and 3) can cause defective embryonic development in neural crest derived structures, in particular melanocytes and enteric nervous systems (ENS) from ET_B_ mutations [18]. Although the importance of ET-3/ET_B_ signalling for migrating neural crest cells of ENS development is well documented, maintaining the pluripotency of neural crest cells, its impact on the development of central nervous system remains unclear [19, 20]. As previously shown, ET_B_ is widely expressed in mammalian brains, including medulla oblongata, cerebral cortex, hippocampus, cerebellum, and striatum [21–24]. Similar distribution is shared by ET-1 and ET-3 [25]. Their co-expressions, therefore, are likely to reflect an undocumented importance.

ET_B_ activation by the endothelin mediates vasoregulation, smooth muscle contractions, neuropeptide transmission, neuronal apoptosis, and potential autonomic control [26–28]. Past studies showed ET_B_ mutations and variants having strong links to hearing deficit, mental retardations, and microcephaly [1, 29]. Concordantly, Yuen et al. (2013) demonstrated marked ET_B_ upregulation in the oligodendrocytes of active multiple sclerosis tissue during remyelination, suggesting an active role in neuromodulation. Further studies using rat cerebellar cultures showed ET_B_ agonist (BQ-3020) and antagonist (BQ-788) promotes and inhibits the remyelination processes, respectively [30]. Although no significant difference in oligodendrocyte number was detected between the control and ET_B_-knockout mice, Swire et al. (2019) reported significantly reduced myelin sheaths in ET_B_-deficient oligodendrocytes. This phenotypically coincided with the decreased sociability among the affected animals [31]. Furthermore, increased neuronal apoptosis have also been detected in the dentate gyrus, hippocampus, and cerebellum of ET_B_ deficient animals [32–34]. These reports therefore suggested ET_B_ exerts a positive impact on neurogenesis.

On the other hand, several reports have suggested ET_B_ inhibition may be beneficial for remyelination, possibly through its regulation on astrocyte proliferation and vascular control. Back et al. (2005) have reported high molecular weight hyaluronan produced by astrocytes can inhibit oligodendrocyte progenitors (OPCs) maturation, which is vital to remyelination post-injury [35]. Consistently, Hammond et al. (2015) reported the inhibition or absence of ET-1/ET_B_ signalling in astrocytes enables oligodendrocyte progenitor cell maturation and promotes remyelination following brain injury [36]. Gadea et al. (2008) also demonstrated the infusion of ET_B_ antagonist, BQ-788, to rat corpus callosum can suppress the upregulations of reactive astrogliosis following acute demyelination injury. Further studies on cell culture suggested this ET-1/ET_B_ activated astrocyte proliferation is mediated by ERK- and JNK- dependent pathways. Inhibitions of these pathways or ET_B_ deficiency may therefore be beneficial to myelination [37]. Furthermore, Grell et al. (2014) and Spray et al. (2017) showed ET_B_ antagonist can suppress cerebral hypoperfusion by inhibiting the vasoconstriction in cerebral arteries. Together, these studies suggested ET_B_^−/−^ mutants may exhibit neuroprotection through the absence of ET_B_ signalling [38, 39]. Given the omnipresence of ET_B_ and its obvious multimodal actions on neural structures and functions, we adopted a macroscopic approach to investigate its effect on brain anatomy and growth.

Prior cell culture studies have suggested possible bimodal actions of ET_B_ on myelination. Although increased neural apoptosis has been reported in ET_B_ deficient animals using immunohistochemistry methodology, to the best of our knowledge, no macroscopic analysis has been completed to evaluate the intracranial changes associated with ET_B_ mutations or HSCR. At most, one anecdotal inference was made from a cohort study showing the regional prevalence of intracranial abnormalities associated with Mowat-Wilson syndrome, a HSCR-associated syndrome caused by mutations in *ZEB2* gene; however, no direct comments have been made on the physical brain size [40]. We aimed to complement previous studies with further anatomical evidence to support ET_B_’s importance on the brain development and to clarify potential correlation between HSCR and intracranial changes.

In this study, we have chosen *sl/sl* rat as the study subject. It is a naturally occurred ET_B_^−/−^ animal model analogous to human WS-IV syndrome with the following characteristics: HSCR, hypopigmentation, and congenital hearing loss [41]. Confirmatory study showed a deletion of 301-bp spanning at the junction between exon1 and intron1 of the ET_B_ gene leading to defective G-protein-coupled receptor, yielding *sl/sl* rat [29, 41]. Segregation analysis from our rat colony data (n = 475) showed *sl/sl* rat follows autosomal recessive inheritance (*p-value* = 0.001) with 95% genetic penetrance for HSCR phenotype, supporting *sl/sl* rat as a good candidate for studying the phenotype of ET_B_ mutation.

Image acquisitions were performed using X-ray micro-computed tomography (micro-CT) and previously described tissue-preparation methods to examine neuroanatomical difference between *sl/sl* rat and the control group [42]. Thorough review on the brain morphology was made and followed by volumetric measurements of selected brain structures. Because neonatal *sl/sl* rat has not reached growth maturity and may suffer HSCR-induced growth impairment, organ growth rate and organ volume/bodyweight ratios were also determined for comparison.

In this study, we hypothesized the following:

1. Gross intracranial morphology may be preserved in ET_B_^−/−^ animals.
2. Reductions in brain and constituent sizes may be presented in ET_B_^−/−^ animals with respect to those of the control group (ET_B_^+/+^ & ET_B_^+/−^). Reducing trends may also extend to the organ growth rate and organ volume/bodyweight ratios of ET_B_^−/−^ animals.
3. Non-uniform anatomical changes associating with homozygous ET_B_ mutation may be presented across different neural constituents; effect of ET_B_ mutation on neuroanatomy may be regional-dependent.

## Methods

### Compliance with ethical practice

All tissues and animals used in this study were handled with strict compliance to both approval bodies: Australian Capital Territory Health-Human Research Ethics Committee (ACTH-HREC) and Australian National University-Animal Experimentation Ethics Committee (ANU-AEEC), approval project number A2011/67.

### Rat culling and method of euthanasia

Rat samples were generated from cross breeding the heterozygous rats (ET_B_^+/−^). The breeding colony was initially derived from a natural-occurring mutation; this colony has been maintained in the Australian National University (ANU) over the past 15 years.

Eleven neonatal rats were sacrificed in this study, with an average age of 88 h. Each rat was anaesthetized with 5% isoflurane for 15 min and culled via abdominal aortotomy. The age, gender, weight, and colonic appearance were recorded prior to staining. Five-millimetre tail tip of each rat was removed and stored for subsequent genotyping.

### Tissue preparation and diffusion staining protocols

Following culling, rats’ heads were isolated from the neck and up in preparation for micro-CT scanning. To ensure successful tissue-staining, a diamond-shaped craniotomy in parietal bone was created through the following: cross-incision of five-millimetre through the skull using a dissection scalpel followed by diagonal excisions of skull using iris scissors.

Post-craniotomy, these tissues were immersed in 10% PBS solution for 30 min to wash out residual body fluid. This was followed by tissue-fixation in 4% formalin solution for 24 h. Next, the formalin was washed out with graded ethanol (EtOH) series over four days: 20%, 50%, 70%, and 90% for 1 day each. These tissue preparations were finalized by seven days of iodine-staining with 1.5% iodine solution in 90% EtOH.

### Image acquisition by micro-CT scanning

All micro-CT datasets of the rats were acquired using a commercially available Caliper Quantum FX in vivo micro-CT scanner. At the expense of 4.5 min scanning time per scan, acquired in vivo micro-CT data has an image-resolution of 20 µm/voxel. The resultant images were stored as DICOM series with average dataset size of 256 MB.

Prior to quantitative analysis, images of in vivo micro-CT scans were validated with two sets of ex vivo micro-CT scans for quality control. The ex vivo micro-CT scans were acquired by scanning two of the eleven rat-tissues using a custom-built micro-CT system at the Applied Mathematic Department of Australian National University. Because ex vivo micro-CT scans have significantly higher signal-to-noise ratio than in vivo micro-CT images, they provided good reference for image quality control; however, accesses to ex vivo micro-CT scanner were limited. Ex vivo micro-CT data with resolution of 10.7 µm/voxel required fifteen hours of scanning time followed by eight hours of image-processing time via Australia National Computational Infrastructure (NCI) services. The resultant images were stored as netCDF files with each dataset size of 12 GB. All datasets were visualized and analysed with FIJI and Drishti, opensource image rendering software [43, 44].

### Image segmentation and volumetric measurements

All acquired in vivo micro-CT raw data were denoised using non-local means (NLM) algorithms to improve signal-to-noise ratio and hence image clarity for segmentation [45]. This code was implemented on a computing server built from Intel (R) Core ™ i7-4770 K CPU @3.5 GHz with 32 GB of RAM and Nvidia GeForce GTX Titan Black Kepler GK110 architecture running Linux.

Following image denoising, micro-CT images were segmented semi-automatically through individual CT-slices for organs of interest using Drishti paint. This segmentation was verified by two operators. A number of neural organs were selected based on prior literature and organ differentiation on micro-CT scan: total brain (TBr), total cerebral cortex (T-CC), total caudate putamen (T-CP), olfactory bulb (OB), medulla (Med), cerebellum (Cer), pituitary gland (Pit), and superior and inferior colliculus (S&I Col). The posterior border of TBr was standardized to 1^st^ cervical spine for comparison across different samples. Cerebral cortex and caudate putamen were segmented and analysed in respective left and right hemispheres prior to amalgamation of measurements. Superior and inferior colliculus were measured together due to poor differentiation between the two regions on micro-CT scans.

Lastly, the segmented data were rendered, visualized, and volumetrically measured with Drishti for volumetric exploration and presentation [44].

### H&E light microscopy

H&E light microscopy was completed for two of eleven rats following micro-CT scanning for verification of neuroanatomical detail presented by micro-CT scans. The tissue processing was conducted in the following steps. The iodine-stained tissues were sectioned longitudinally into blocks of 4 mm in thickness and placed in cassettes. Contrast washout and dehydration were then performed in 90% EtOH for 48 h prior to paraffin embedment at 60 °C. Next, the samples were sliced with a microtome for tissue-sheets of 4 µm in thickness. These tissue-sheets were then laid in water bath of 5–6 °C while being positioned onto labelled-glass slides. These slides were dried overnight at 37 °C.

Progressive H&E staining was then performed. The slides were placed in alum-hematoxylin solutions until the visualization of dark red colour. This was followed by washing and ‘bluing’ with lithium carbonate solution. Finally, the slides were washed before counter-staining with 0.5% eosin alcoholic solution.

All H&E slides were examined with an Olympus IX71 microscope with 4 × magnification.

### Genotyping

Genotyping was completed following the completion of the quantitative image analysis of rat data to avoid observational bias. It was completed by standard PCR methods. Three wild types (ET_B_^+/+^), three heterozygotes (ET_B_^+/−^), and five *sl/sl* (ET_B_^−/−^) rats were identified. The average ages of wild-type, heterozygotes, and *sl/sl* groups were 90.7 h, 96 h, and 83.2 h, respectively.

PCR genotyping was processed through the following. Five-millimetre tail tips from each of the eleven rats were isolated for lysis using Proteinase K in lysis buffer, which consisted of 100 mM Tris pH 8, 5 mM EDTA, 0.2% SDS, and 200 mM NaCl in distilled water. The DNA was extracted via vortex heating and centrifuging to separate DNA-containing supernatant from the undigested materials. Afterwards, the supernatant was further vortexed and centrifuged to isolate the DNA pellet, followed by 70% EtOH washing and drying. The DNA was then suspended and quantified with spectrophotometry. Next, PCR was completed with “Master-Mix” reagent: 10*PCR buffer Qiagen-contained MgCl2, dNTP (10 mM), Primer PS7 (33.3 μM; 5’-CCA CTA AGA CCT CCT GGA CT-3’), Primer PS15 (33.3 μM; 5’-TCA CGA CTT AGA AAG CTA CAC T-3’) and DNA polymerase [46]. The Master-Mix reagent was then pipetted onto PCR plates with fourteen rat DNAs (including the known controls) and placed in Veriti 96-Well Thermal-Cycler for PCR. Lastly, electrophoresis was run at 100 V and 55 mA for 1 h. Eleven test-subject DNAs were run against positive controls for wild-type, heterozygote, and *sl/sl* with molecular weight marker (MassRuler, #SM1263, Fermentas) by the side. Electrophoresis gel was visualized with Gel Documentation System DOC-Print VX5 (Vilber Lourmat).

### Comparison and Statistical analysis

Based on segregation analysis on cumulative colony data showing *sl/sl* rat phenotype followed autosomal recessive inheritance (*p-value* = 0.001) with high genetic penetrance, up to 95% of *sl/sl* rat exhibited HSCR, statistical comparisons were made between *sl/sl* (ET_B_^−/−^) and the control group (ET_B_^+/+^ & ET_B_^+/−^) to determine the effect of ET_B_ on structural changes and growth. This approach was consistent with the autosomal recessive inheritance of human WS-IV, a disease caused by ET_B_^−/−^ genotype. Nonetheless, we have also performed one-way ANOVA analysis and post hoc Tukey to assess the potential relations between ET_B_ gene dose and the following selected parameters.

The parameters included for comparison were as follows: bodyweight (g), bodyweight growth rates (g/Hr), organ volume (mL), organ volume growth rates (mL/Hr), and organ volume/bodyweight ratios (mL/g). The bodyweight growth rate (g/Hr) was calculated by dividing each rat’s bodyweight (g) to its respective age (Hr) at culling and followed by statistical comparison between the control and mutant groups. Similarly, organ volume growth rate (mL/Hr) and organ volume/bodyweight ratios (mL/g) were calculated from respective volumetric measurements (mL), age (Hr), and bodyweight (g) at times of culling.

Standardized comparisons of organ volume growth rate and organ volume/bodyweight ratios were made to exclude potential confounding factors of age and body-size variance. Additionally, these measures provided estimating metrics for the organ changes upon developmental maturation of the animals. Moreover, the proportionality of individual neural substituent with respect to the total brain (organ/total brain) was compared to explore potential regional-dependent effect of ET_B_. All parameters were expressed as mean ± sem in figures. The relative parametric difference (%) between *sl/sl* and the control group was also calculated and reported throughout this study for better appreciation on the degrees of difference.

## Result

### ET_B_^−/−^ mutant has lower bodyweight and body growth rate

**Fig. 1:**
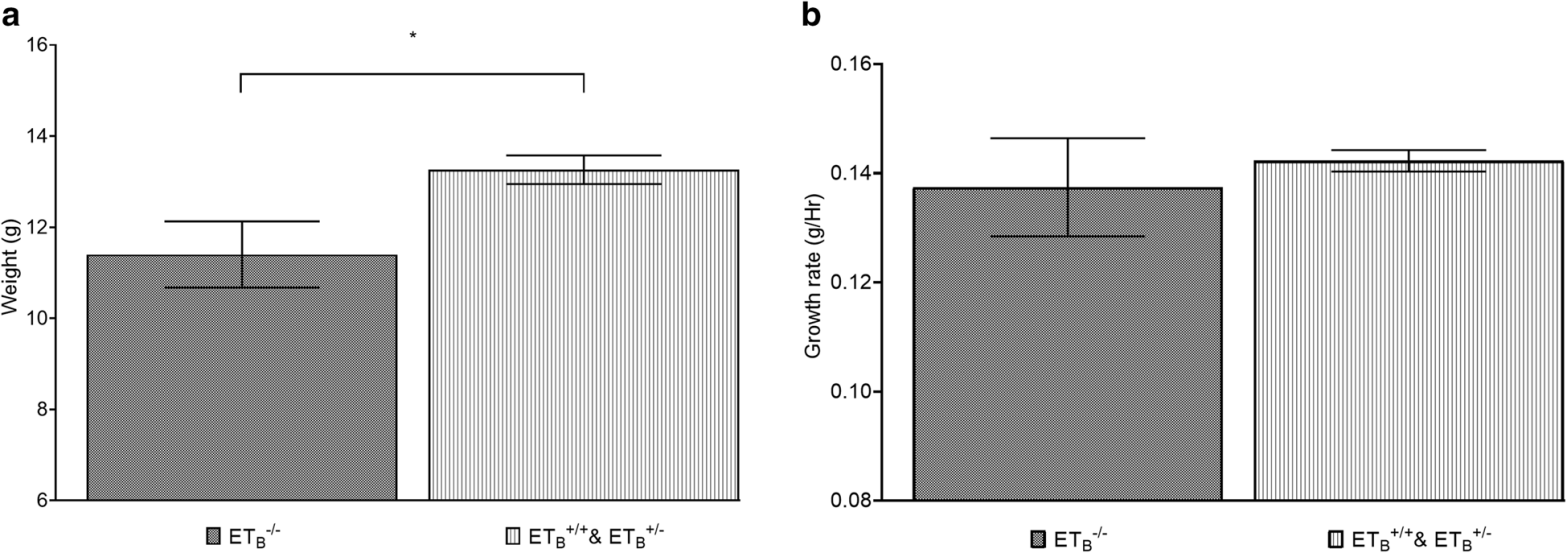
Homozygous ET_B_ mutation was associated with reductions in bodyweight (**A**) and bodyweight growth rate (**B**).* sl/sl* (ET_B_^−/−^) rat has a significantly lower bodyweight than that of the control group (ET_B_^+/+^ & ET_B_^+/−^), 11.40 g versus 13.26 g (df = 9, t-value = 2.51, *p-value* = 0.03), **A**. A similar but smaller difference was observed in the comparison of body growth rate, 0.137 g/Hr versus 0.142 g/Hr (df = 9, t-value = 0.58, *p-value* = 0.58), **B**. This suggested *sl/sl* rat was likely to have a proportional organ size shrinkage; furthermore, reduced body growth rate suggested developmental impairment may continue to worsen until maturity. *statistically significant, p-value  ≤  *0.05*

We compared the bodyweight of the studied animals to evaluate potential growth reduction associated with ET_B_^−/−^ mutation. As shown by Fig. 1A, *sl/sl* rat has 16.32% lower bodyweight than the control group, *p-value* = 0.03. Although not reaching statistical significance, a 3.53% deduction in growth rate was also detected, Fig. 1B. Both findings supported potential impairments in global body growth. On the other hand, one-way ANOVA demonstrated no significant variability in bodyweight or body growth rate among the three genotypes, as shown by Additional file 1: Table S1. These observations suggested ET_B_ mutation may be associated with body growth impairment in an autosomal recessive manner.

### Gross intracranial morphology was overall preserved in ET_B_^−/−^***mutant***

**Fig. 2:**
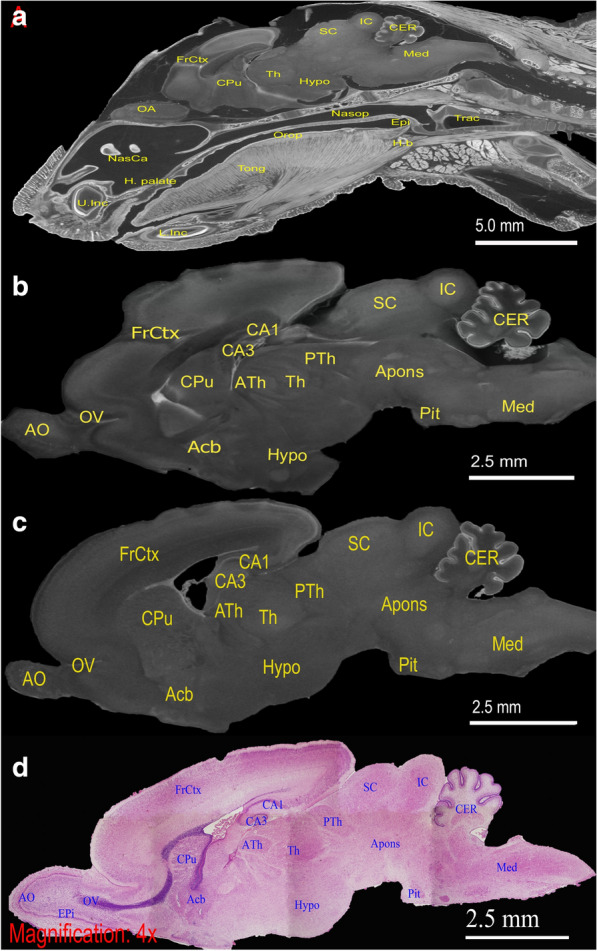
ET_B_^−/−^ rat has a grossly normal intracranial morphology.** A**, and **B** represented respective parasagittal micro-CT views of intracranial morphology and isolated brain of *sl/sl* (ET_B_^−/−^) rat. **A** demonstrated the presence of major craniofacial features and neural structures as labelled. **B** illustrated magnified micro-CT view of the ET_B_^−/−^ brain with additional details, which were also presented in the micro-CT scans of wildtype (ET_B_^+/+^) rat, **C**. Lastly, H&E micrograph of the micro-CT scanned *sl/sl* rat brain confirmed the presence of the identified structures, **D**. The anatomical features identified were labelled as follows: *Acb*  accumbens nucleus, *AO*  anterior olfactory bulb, *apons*  anterior pons, *CA1*  CA1 field of hippocampus, *CA3*  CA3 field of hippocampus, *CER*  cerebellum, *Cpu * caudate putamen, FrCtx  frontal cortex, *Hypo*  hypothalamus, *IC*  inferior colliculus, *med*  medulla, *Pit*  pituitary gland, *PTh*  posterior thalamus, *SC * superior colliculus, *Th*  thalamus, *U*. Inc  upper incisor, *Tong*  tongue, *Trac*  trachea, *Epi * epiglottis, *H. b*  hyoid bone, *H. palate*  hard palate, *L.* Inc  lower incisor, *NasCa*  nasal cavity, *Nasop*  nasopharynx, *Orop*  oropharynx

Previous study has suggested disruptions in the endothelin system can lead to cephalic neural crest cell (CNCC) migration failure and thus marked craniofacial defects [47]. However, thorough reviews of *sl/sl* rat scan (Fig. 2B) demonstrated grossly normal cranial morphology, similar to that of wild type rat (Fig. 2C). As shown by the parasagittal view of micro-CT images and H&E microscopy, Fig. 2B – D, *sl/sl* rat brain contained all the major components as the control group: accumbens nucleus, anterior olfactory bulb, anterior pons, hippocampus, cerebellum, caudate putamen, frontal cortex, hypothalamus, inferior colliculus, medulla, pituitary gland, posterior thalamus, superior colliculus, and thalamus. Additionally, review of facial anatomy did not reveal obvious disfiguration, as shown by Fig. 2A. These suggested intracranial impairments associated with ET_B_ mutation, if any, may be subtle and limited to size variance rather than constituent agenesis or morphological changes.

### Homozygous ET_B_ mutation was associated with significant decreases in brain size

**Fig. 3:**
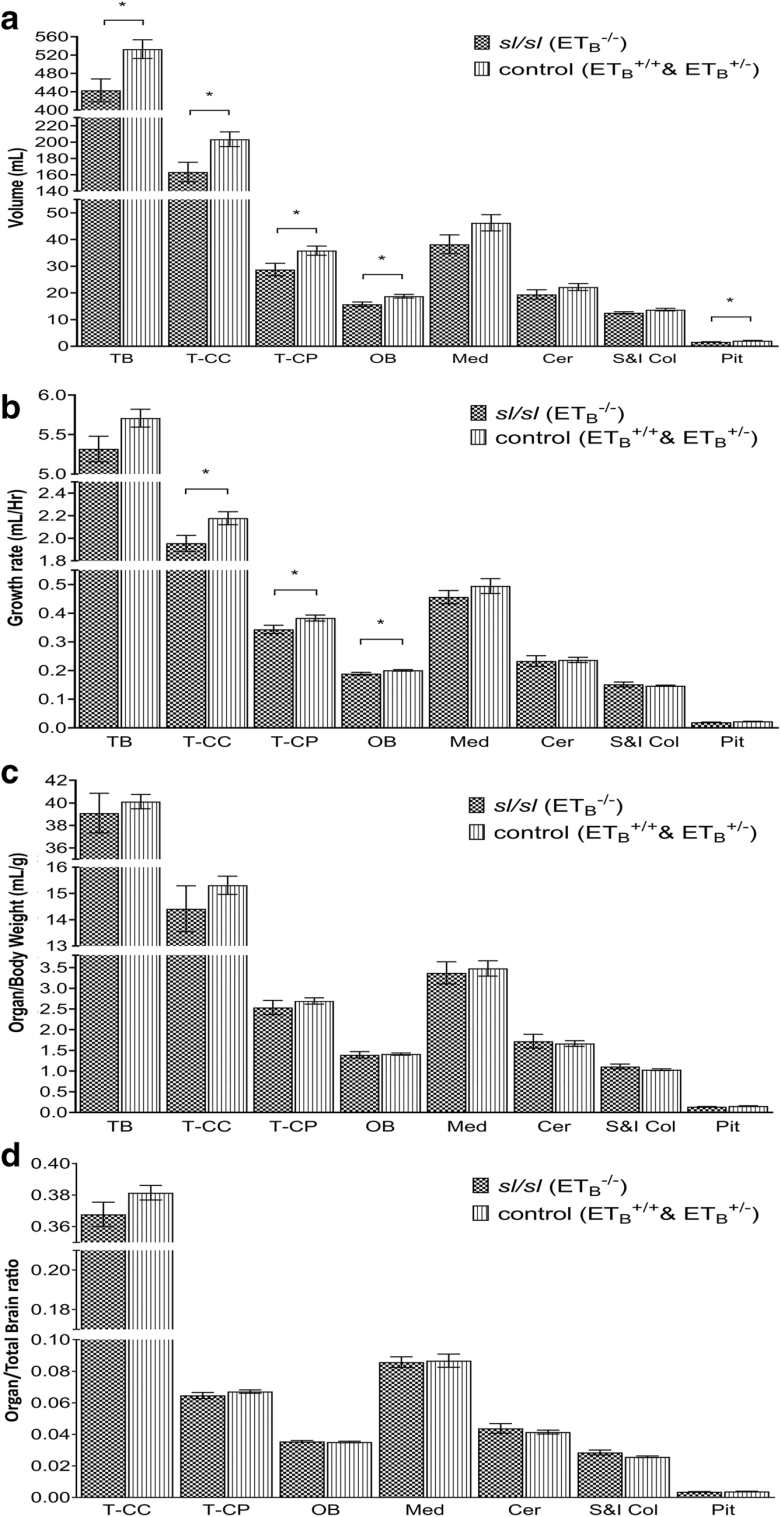
ET_B_^−/−^ mutant has reduced neural organ volume, organ growth rate, organ volume/bodyweight indices, and organ/total brain ratios. **A** Significant volumetric reductions in brain and constituents were detected in young ET_B_^−/−^ animals. *sl/sl* rat has statistically smaller TB than the control rat: 443.02 mm^3^ versus 533.12 mm^3^ (df = 9, t-value = 2.82, *p-value* = 0.02). Similar trend was extended across the following: T-CC (163.36 mm^3^ versus 203.55 mm^3^, df = 9, t-value = 2.73, *p-value* = 0.02), T-CP (28.75 mm^3^ versus 35.84 mm^3^, df = 9, t-value = 2.49, *p-value* = 0.03), OB (15.73 mm^3^ versus 18.74 mm^3^, df = 9, t-value = 2.78, *p-value* = 0.02), and Pit (1.60 mm^3^ versus 2.09 mm^3^, df = 9, t-value = 2.95, *p-value* = 0.02). While not reaching the statistical power, measurements of Med (38.22 mm^3^ versus 46.27 mm^3^, df = 9, t-value = 1.74, *p-value* = 0.12), Cer (19.43 mm^3^ versus 22.19 mm^3^, df = 9, t-value = 1.30, *p-value* = 0.23), and S&I Col (12.51 mm^3^ versus 13.73 mm^3^, df = 9, t-value = 1.84, *p-value* = 0.10) also demonstrated detectable reduction in *sl/sl* rats. **B **ET_B_^−/−^ rat has significantly lower growth rates across different parts of brain. Lower TBr growth rate was observed in *sl/sl* rat with respect to the control animal, 5.32mm^3^/Hr versus 5.71mm^3^/Hr (df = 9, t-value = 2.03, *p-value* = 0.07). Consistent difference between the two groups was also seen in the following: T-CC (1.96mm^3^/Hr versus 2.18mm^3^/Hr, df = 9, t-value = 2.46, *p-value* = 0.04), T-CP (0.34mm^3^/Hr versus 0.38mm^3^/Hr, df = 9, t-value = 2.26, *p-value* = 0.05), OB (0.19mm^3^/Hr versus 0.20mm^3^/Hr, df = 9, t-value = 2.23, *p-value* = 0.05), Med (0.46mm^3^/Hr versus 0.50mm^3^/Hr, df = 9, t-value = 1.10, *p-value* = 0.30), and Pit (0.019mm^3^/Hr versus 0.022mm^3^/Hr, df = 9, t-value = 2.11, *p-value* = 0.06). On the other hand, minimal difference between the two groups was detected in the following: Cer (0.23mm^3^/Hr versus 0.24mm^3^/Hr, df = 9, t-value = 0.19, *p-value* = 0.85) and S&I Col (0.15mm^3^/Hr versus 0.15mm^3^/Hr, df = 9, t-value =  − 0.55, *p-value* = 0.60). **C **ET_B_^−/−^ rat has lower neural organ/bodyweight ratios, suggesting intrinsic ET_B_ effects on brain development. *sl/sl* rat has lower a TBr/bodyweight ratio than that of the control group, 39.1mm^3^/g versus 40.12mm^3^/g (df = 9, t-value = 0.58, *p-value* = 0.57), reflecting a disproportionally larger reduction in TBr with respect to changes in its bodyweight. Similar trends were also showed in the following: T-CC (14.42mm^3^/g versus 15.31 mm^3^/g, df = 9, t-value = 1.02, *p-value* = 0.34), T-CP (2.53mm^3^/g versus 2.69 mm^3^/g, df = 9, t-value = 0.91, *p-value* = 0.39), OB (1.39mm^3^/g versus 1.41mm^3^/g, df = 9, t-value = 0.23, *p-value* = 0.82), Med (3.37mm^3^/g versus 3.48mm^3^/g, df = 9, t-value = 0.34, *p-value* = 0.74), and Pit (0.14mm^3^/g versus 0.16mm^3^/g, df = 9, t-value = 2.00, *p-value* = 0.08). On the other hand, *sl/sl* rat has slightly larger indices in the following: Cer (1.72mm^3^/g versus 1.67mm^3^/g, df = 9, t-value =  − 0.32, *p-value* = 0.75) and S&I Col (1.11mm^3^/g versus 1.03mm^3^/g, df = 9, t-value =  − 1.29, *p-value* = 0.23). Overall, ET_B_ mutation was associated with more marked changes in the neural organs. In addition, variability in the difference of organ/bodyweight ratios between *sl/sl* rat and the control group suggested that the effect of ET_B_ mutation maybe spatially dependent within the brain. **D** Disproportional changes in the neural organ/total brain indices of ET_B_^−/−^ rat suggested non-uniform ET_B_ effect in different parts of the brain**. **Subtle disproportional changes in neural constituents with respect to the global brain shrinkage were detected in sl/sl rat. Reduced organ/total brain ratios were seen in the following structures of sl/sl rat: T-CC (0.37 versus 0.38, df = 9, t-value = 1.58, *p-value* = 0.15), T-CP (0.065 versus 0.067, df = 9, t-value = 1.17, *p-value* = 0.27), Med (0.086 versus 0.087, df = 9, t-value = 0.17, *p-value* = 0.87), and Pit (0.0036 versus 0.0039, df = 9, t-value = 1.54, *p-value* = 0.16). On the contrary, organ/total-brain indices of OB (0.036 versus 0.035, df = 9, t-value = − 0.54, *p-value* = 0.60), Cer (0.044 versus 0.041, df = 9, t-value = − 0.75, *p-value* = 0.47) and S&I Col (0.029 versus 0.026, df = 9, t-value = − 1.78, *p-value* = 0.11) were larger in *sl/sl* rats. This variation in changes suggested that ET_B_ effect may be regional dependent within the brain. *TB* Total Brain, *T-CC* Total Cerebral Cortex, *T-CP * Total Caudate Putamen, *OB*  Olfactory Bulb, *Med*  Medulla, *Cer*  Cerebellum, *S&I Col*  Superior and Inferior Colliculus, *Pit*   Pituitary gland. *statistically significant, *p-value*  ≤  0.05

While the gross morphology might be preserved, moderate volumetric reductions of the brain and constituents were detected in *sl/sl* mutants. As demonstrated by Fig. 3A, a significant reduction of 20.33% was observed in the brain of *sl/sl* rat, *p-value* = 0.02. Furthermore, an average of 20% volumetric reduction was detected across all neural constituents analysed in the *sl/sl* rat. Structures with statistically significant difference between *sl/sl* and the control groups were as follow: T-CC (24.60%, *p-value* = 0.02); T-CP (24.68%, *p-value* = 0.03); OB (19.13%, *p-value* = 0.02); Pit (30.08%, *p-value* = 0.02). Although not reaching the statistical power, the reducing trend in *sl/sl* rat was also evident in the following: Med (21.05%, *p-value* = 0.11), Cer (14.22%, *p-value* = 0.22); S&I col (9.73%, *p-value* = 0.10).

Additionally, one-way ANOVA showed statistically significant variation in TB, T-CC, and Pit volumes of the three genotypes, as shown by Additional file 2: Table S2A. However, post hoc Tukey revealed significant differences were only presented between the *sl/sl* and heterozygous group; consistent ET_B_ dose-dependent trend was not seen in the wild type, as shown by Additional file 2: Table S2B.

### Homozygous ET_B_ mutation was associated with significantly lower intracranial growth rate

Reduced intracranial organ growth rate was observed in *sl/sl* group; however, this margin of reduction was smaller than that detected in the volumetric changes. An approximately 10% reduction in growth rate was observed across various brain components in *sl/sl* rat.

As shown by Fig. 3B, the TBr growth rate of *sl/sl* rat was 7.35% lower than that of the control group, *p-value* = 0.07. Consistent decreasing trends were also presented in the following: T-CC (11.42%, *p-value* = 0.04), T-CP (11.51%, *p-value* = 0.05), and OB (6.21%, *p-value* = 0.05). Furthermore, although not reaching the statistical power, similar reductions were observed in the medulla (8.48%, *p-value* = 0.30) and pituitary gland (15.62%, *p-value* = 0.06) of *sl/sl* rat. Unexpectedly, no significant change was detected in the growth rate of Cer (1.60%, *p-value* = 0.85) and S&I Col (2.72%, *p-value* = 0.60). Lastly, no consistent linear relationship was detected between the neural growth rates and ET_B_ gene dose, as shown by one-way ANOVA analysis in Additional file 3: Table S3A.

Overall, we found homozygous ET_B_ mutation was associated with reduced neural growth rate during development, an effect likely to persist until maturation age.

### Homozygous ET_B_ mutation was associated with lower brain volume/bodyweight indices

*sl/sl* rat has disproportionally larger reductions in its brain volumes than the decreases in its bodyweight. As illustrated by Fig. 3C, *sl/sl* rat has lower organ volume/bodyweight ratios than the control group: TBr (2.60%, *p-value* = 0.57), T-CC (6.21%, *p-value* = 0.15), T-CP (6.30%, *p-value* = 0.27), OB (1.28%, *p-value* = 0.60), Med (3.22%, *p-value* = 0.87), and Pit (11.18%, *p-value* = 0.08). Albeit statistically underpowered, these findings suggested neural development was more sensitive to ET_B_ dysfunction than the bodily growth. And although homozygous ET_B_ mutation seemed to predominantly affect growth negatively, *sl/sl* rat was found to have larger organ/bodyweight ratios in its Cer (3.17%, *p-value* = 0.47) and S&I Col (6.84%, *p-value* = 0.23). This raised the possibility that a potential protective mechanism associated with ET_B_^−/−^ may be presented, albeit this protective effect was likely small. Additionally, non-uniform changes were recorded from different neural structures of *sl/sl* rat, suggested the impact of ET_B_ mutation on anatomical development might be dependent on organ location.

### ET_B_^−/−^ mutant has subtle and non-uniform changes across different neural constituents/total brain indices

As showed by Fig. 3D, *sl/sl* rat and the control group shared subtle difference between their respective neural organ/TBr indices. Although these differences may be small, ranging from 2 to 8%, they reflected disproportional changes in different neural constituents of ET_B_^−/−^ rat. These variations suggested that the effect of ET_B_ on structural growth may be influenced by the target organ locations within the brain.

## Discussion

As evidenced by the anatomical changes detected, we supported the notion that homozygous ET_B_ mutation and associated HSCR were likely linked with intracranial changes, at least in the sense of volumetric measurements. This finding was consistent with previous in vitro studies and clinical reports citing HSCR’s associations with neurological deficits, as exemplified by Down’s syndrome, Haddad syndrome, Mowat-Wilson syndrome, and WS-IV [1, 7, 48–51].

It has been suggested that disruption in ET-1/ECE/ET_A_ pathway system can lead to marked craniofacial dysmorphism. [47]. And agenesis of corpus callosum and hippocampus have also been reported in HSCR associated syndromes [50, 52]. Regardless, these gross abnormalities were not detected in *sl/sl* rat, as demonstrated by Fig. 2. This observation suggested that while ET_B_ mutation may disrupt enteric neural crest cell migration, its direct impact on the migration of cephalic and vagal neural crest precursors may be limited. On the other hand, neurogenic modelling mediated by ET_B_ signalling and the clinical reporting of HSCR-associated mental retardation supported the notion that ET_B_ may possess an adjunct but important role in neural development [3, 53]. Quantitative analysis on the neuroanatomy was therefore an intuitive investigation.

Many previous studies have demonstrated the presence of ET_B_ receptors in olfactory bulb, cerebral cortex, caudate putamen, thalamus, hypothalamus, pituitary gland, brain stem and limbic system through the combined use of immunohistochemistry and ET_B_’s agonist or antagonists, supporting its importance in brain structures and functions [54–57].

Consistently, two-dimensional (2D) immunofluorescence studies have demonstrated ET_B_ stimulation by IRL-1620 can lead to neurogenic remodelling, which was likely achieved by increased neurogenesis and angiogenesis via elevated vascular endothelial growth factor (VEGF) and nerve growth factor (NGF) [2, 3, 58]. Additionally, Feger et al. (1997) reported that activation of endothelial ET_B_ by ET-1 can trigger vasodilation of basilar artery through the production of nitric oxide [59], thereby improving cerebral perfusion. Consequently, these studies provided the supports that ET_B_ activation promotes neuroprotection. This was indeed consistent with the overall brain size reduction observed in *sl/sl* rat, which lacks functional ET_B_ during its development. On the other hand, in vitro studies using U0126, a mithramycin A (MitA) or mitogen-activated protein kinase (MEK) 1/2 inhibitor, blocks the elevation of ET_B_-mediated vasoconstriction following cerebral ischemic events, suggesting ET_B_ stimulation in vascular smooth muscle cells (VSMC) may worsens neural damage [38, 60, 61] by triggering further cerebral hypoperfusion. These conflicting findings suggested the effect of ET_B_ on brain growth may be dynamic and regional-dependent, a pattern consistent with our finding, Fig. 3D, showing variability in organ changes with respect to global brain size reduction.

Despite the presentation of a grossly normal brain morphology, a significant structural reduction of 20.33% (*p-value* = 0.02) was found in the brain volume of *sl/sl* rat at the age of 88 h. Consistently, a reduction of 7.35% (*p-value* = 0.07) in brain growth rate also suggested its structural impairment would likely worsen upon developmental maturity. Several factors could have contributed to these impairments. As previously reported by McDougall et al. [62], intrauterine growth restriction can alter the postnatal cerebellum development [62]. While we have not detected an underdevelopment in the birthweight of *sl/sl* rat based on our colony data of 864 rats, we did record a bodyweight reduction of 16.32% at the age of 88 h in our studied rats, Fig. 1. This was consistent with the fact that adequate maternal uterine perfusion through functional ET_B_-mediated vasodilation can be achieved in the heterozygous parents of *sl/sl* rat [63]; however, postnatal growth of *sl/sl* rat may later suffer the negative effect of HSCR-induced malnutrition and ET_B_^−/−^-mediated mesenteric hypoperfusion [1, 64]. While the brain shrinkage in the *sl/sl* rat can be partially explained by this bodily growth impairment, disproportionally larger reductions in *sl/sl* rat brain with respect to changes in its bodyweight, as shown by Fig. 3C, suggested additional intrinsic ET_B_ mechanisms on the brain development. Indeed, this was supported by *sl/sl* rat exhibiting 1.26% to 11.18% smaller organ volume/bodyweight indices in TBr, T-CC, TCP, OB, Med, and Pit. Interestingly, opposite trend was seen in Cer and S&I Col measurements, with *sl/sl* rat having 3.17% and 6.84% higher indices, respectively. In conjunction with ET_B_’s functional duality on vasoregulation and neurogenesis reported previously, our observations likely reflected a potential regional-specific neuroprotection by ET_B_ mutation despite its overwhelming negative effects [3, 32–34, 36, 38, 58, 59].

Dysregulation in the neurogenesis, angiogenesis, and cerebral vascular tone have likely contributed to the observed structural changes in *sl/sl* rat. This may be mediated directly by the ET_B_ expression on neuroblasts or neural tissue, which promotes cell division and inhibits apoptosis, or by indirect mechanisms including vascular growth stimulation and vasospasm inhibition. Indeed, Nishikawa et al. (2011) showed ET_B_-stimulation by IRL-1620 accelerates interkinetic nuclear migration (INC) of cortical neural progenitor cells (NPC), whereas inhibition by BQ-788 results in INC deceleration and subsequent mislocalization of NPC [65]. Additionally, Koyama et al. (2011) demonstrated intracerebroventricular ET_B_ stimulation increases vascular endothelial growth factor A (VEGF-A), thereby triggering VEGF-receptor signalling in cerebrum but not hippocampus. This suggested regional-dependent neuroprotective effect of ET_B_, possibly via vascular control [66]. Moreover, the loss of ET_B_-mediated clearance caused significant elevation of ET-1 in *sl/sl* rat, thus leading to ET-1/ET_A_-mediated hyper-vasoconstriction in cerebral vasculature [67]. Albeit minor, endothelial-mediated basal vasodilation from ET-1/ET_B_ stimulation was also lost in *sl/sl* rat [4, 68]. Together, these processes retarded the cerebral growth through impaired neurogenesis and cerebral perfusion. Conversely, despite findings of overall size reduction in majority of neural constituents of *sl/sl* rat, small elevation in Cer/bodyweight and S&I Col/bodyweight indices lend some support to the argument that regional brain development may benefit from increased perfusion secondary to reduced ET_B_-mediated vasoconstriction, for instance, in the VSMC of collicular artery [38]. However, this mutation-associated beneficial effect was likely minor in comparison to the dominant ET-1/ET_A_ effect [38, 61, 69].

If our finding was extrapolatable to human subjects, then the observed structural impairments lend supports to several HSCR-associated clinical anomalies. For instance, marked reductions in total brain (20.33%), cerebral cortex (24.60%), and caudate putamen (24.68%) were not only consistent with prior observations of mental delay in some HSCR patients and but also raised the questions that these patients may be more susceptible to Parkinson’s disease or depression secondary to potential dopamine deficiency [70, 71]. Indeed, Webber’s et al. (1998) have demonstrated that ET-1 stimulation to rat striatum via activation of ET_B_ triggers the release of dopamine [22]. Moreover, detection of medulla shrinkage (21.05%) provided some anatomical supports for dysregulated autonomic functions associated with some HSCR patients, as exemplified by reports of Haddad syndrome, congenital central hypoventilation syndrome, and cardiac arrhythmias [51, 72]. In addition, volumetric reduction in cerebellum, up to 14.22%, supported clinical reports of ataxia observed in WSI-IV patient [12]. This was also consistent with increased neural apoptosis and reduced neural proliferations detected in the cerebellum of *sl/sl* rat [32, 34]. Lastly, size reductions in olfactory bulb (19.13%) and pituitary gland (30.08%) of *sl/sl* rat were consistent with the ET_B_ distribution [21–24, 73]. While there was no distinctive clinical syndrome currently described to associate HSCR with pituitary dysfunctions, increased risks for familial medullary carcinoma (FMTC) and multiple endocrine neoplasia type II (MEN II) in these patients may be linked to the pituitary structural defects [74–76]. It was again worth noting that these reported measurements were made on immature animals, and as suggested by the growth rate discrepancy illustrated in Fig. 3B, structural defects in ET_B_^−/−^ individuals were therefore likely to worsen. Hence, more prominent structural impairments were likely to reflect HSCR-associated functional declines upon maturity.

Lastly, we compared the structural differences among the three genotypes to explore potential dose-dependent effect of ET_B_. While heterozygote has significantly larger TB, TCC and Pit than those of *sl/sl* rats, no linear relationship can be drawn from its comparison with the wild-type rat, Additional file 2: Table S2B. We suspected statistical under-power was the likely explanation. However, we also acknowledged that, in conjunction with some reports, our observation was partially consistent with the notion that a single functional ET_B_ gene may be sufficient to prevent neuroapoptosis and mediate ET-1 clearance in some structures, thereby improving cerebral perfusion and exerting some neuroprotection [2, 3, 38, 58, 60, 61, 67]. Overall, it was of little doubt that homozygous ET_B_ mutation impairs brain structures. Our findings were consistent with ET_B_ mutation acting as an autosomal recessive condition with high penetrance.

In summary, we provided quantitative 3D evidence illustrating structural changes in the brain associating with HSCR, at least in ET_B_^−/−^ animal model. These detrimental effects were not uniform across different brain structures and minor protective effects may be presented, Fig. 3C, D, potentially through reduced cranial vessel tone. Nevertheless, overall findings remained consistent with several proposed mechanisms of action by ET_B_, including regulations on angiogenesis, neurogenesis and vasoconstriction [2, 3, 38, 58, 60, 61, 65, 66, 69]. Our results supported functional ET_B_s have predominantly neuroprotective benefits during neonatal development.

Although we have shown ET_B_ mutation affects brain growth, further study was warranted. We acknowledged a larger sample size would be beneficial along with more efficient image-analysing software to improve statistical power; nevertheless, current finding cannot be overlooked. More importantly, given that we have macroscopically identified ET_B_-affected neural organs, future cell proliferation studies using 5-bromo-2’-deoxyuridine (BrdU) labelling can be useful in confirming reduced neural proliferation microscopically [34]. Moreover, this can also be supplemented by the measurements of VEGF to confirm the angiogenesis reduction in *sl/sl* rats [2]. Lastly, confirmatory study on basilar arterial dilatation using pressure myography can clarify vasoprotective mechanism mediated by ET_B_ [39].

## Conclusion

Despite exhibiting a grossly normal cranial morphology, an average of 20% volumetric reduction was detected in the brain and constituents of ET_B_^−/−^ HSCR model. With no significant differences between wild-type and heterozygous animals, these changes associated with ET_B_ mutation were consistent with autosomal recessive inheritance. The non-uniform neuroanatomical changes observed across different constituents supported prior studies on differential ET_B_ distribution, neurogenesis, and vasoregulation in the brain. Interestingly, a regional-dependent protective effect of ET_B_ may be presented, potentially corresponding to the circulatory distribution through ET-1/ET_B_-mediated vasoregulation. Overall, our model findings supported the notion that ET_B_^−/−^ HSCR patients may exhibit neuroanatomical impairments, consistent with prior clinical reports. Although further study involving human subjects was encouraged, this study supported the importance to a more comprehensive management plan for HSCR patients.

## Supplementary Information

**Additional file 1: Table S1:** No significant variation was detected in the mean bodyweight (p = 0.0551) or mean body growth (p = 0.9096) of the studied rats, which consisted of three genotypes: ET_B_^+/+^, ET_B_^±^, and ET_B_^−/−^. This suggested bodily growth restriction associated with ET_B_ mutation, if presented, was not a dose-dependent relationship.

**Additional file 2: Table S2:** One-way ANOVA showed statistically significant variations in the TBr, TCC, and Pit volumetric means of the studied rats: ET_B_^+/+^, ET_B_^+/−^, and ET_B_^−/−^. On the contrary, no significant variation was detected in other organ measurements of the three genotypes, as shown by **A** Post-hoc Tukey showed significant volumetric difference between the heterozygote (ET_B_^+/−^) and *sl/sl* (ET_B_^−/−^) in TBr (mean difference 113.7, SE 38.02, p = 0.0412), TCC (mean difference 52.07, SE 17.24, p = 0.0394), and Pit (mean difference 0.6113, SE 0.1927, p = 0.0315). However, measurement of wild type (ET_B_^+/+^) was not significantly different from those of the other two groups, as shown by **B.**

**Additional file 3: Table S3:** One-way ANOVA comparison showed no significant difference was detected in the mean organ growth (**A**), volumetric/bodyweight ratios (**B**), and organ/whole-brain ratios (**C**) of the three genotype rats: ET_B_^+/+^, ET_B_^+/−^, and ET_B_^−/−^. These findings suggested that effect of ET_B_ mutation was likely non-linear to gene doses.

## Data Availability

Full measurement datasheet is available in Additional File. All original rat micro-CT files are stored in the Canberra data centre of National Computational Infrastructure (NCI) Australia for security and processing. Original image files are available upon request. Request may be sent directly to corresponding author at ckochin@gmail.com.

## Abbreviations

ANU-AEEC: Australian National University Animal Experimentation Ethics Committee
ACTH-HREC: ACT Health Human Research Ethics Committee
BrdU: 5-Bromo-2’-deoxyuridine
CC: Cerebral cortex
CER: Cerebellum
CNS: Central nervous system
CP: Caudate putamen
Df: Degree of freedom
DICOM: Digital imaging and communications in medicine
DNA: Deoxyribonucleic acid
GDNF: Glial cell line derived neurotrophic factor
GFRα1: GDNF family receptor alpha 1
GI: Gastrointestinal
H&E: Hematoxylin and eosin stain
HSCR: Hirschsprung’s disease
INM: Interkinetic nuclear migration
ECE: Endothelin converting enzyme
ECE-1: Endothelin converting enzyme-1
ENS: Enteric nervous system
ET-1: Endothelin-1
ET-3: Endothelin-3
ET_A_: Endothelin-A receptor
ET_B_: Endothelin-B receptor
EtOH: Ethanol
Med: Medulla
MEK: Mitogen-activated protein kinase
MitA: Mithramycin A
Micro-CT: Micro-computed tomography
mRNA: Messenger ribonucleic acid
NCC: Neural crest cells
NCI: National Computational Infrastructure
NGF: Nerve growth factor
NLM: Non-Local Means
NPC: Neural progenitor cells
NTN: Neurturin
OB: Olfactory bulb
PCR: Polymerase chain reaction
Pit: Pituitary gland
RET: Proto-oncogene RET
S&I Col: Superior and inferior colliculus
*sl/sl*: Spotting-lethal
SOX10: Sry-related HMg-Box gene 10
TBr: Total brain
3D: Three-dimensional
2D: Two-dimensional
VEGF: Vascular endothelial growth factor
VEGF-A: Vascular endothelial growth factor-A
WS-IV: Waardenburg-Shah syndrome
