# Supplementary material for: Brain size reductions associated with endothelin B receptor mutation, a cause of Hirschsprung’s disease
Source: BMC Neurosci. 2021 Jun 19;22:42. doi: 10.1186/s12868-021-00646-z (PMC8214790; doi:10.1186/s12868-021-00646-z)
Supplement: Supplementary file 1 — Additional file 1: Table S1: No significant variation was detected in the mean bodyweight (p = 0.0551) or mean body growth (p = 0.9096) of the studied rats, which consisted of three genotypes: ETB+/+, ETB±, and ETB−/−. This suggested bodily growth restriction associated with ETB mutation, if presented, was not a dose-dependent relationship. [file 12868_2021_646_MOESM1_ESM.docx]

| Supplementary Table1 : One-Way ANOVA for Body Parameters | | | | | |
| --- | --- | --- | --- | --- | --- |
|  | Sum of Squares | df | Mean Square | F | Significance |
| Body Weight | | | | | |
| Between Groups | 10.40 | 2 | 5.201 | F (2,10) = 3.927 | P=0.0551 |
| Within Groups | 13.24 | 10 | 1.324 |  |  |
| Total | 23.64 | 12 |  |  |  |
| Body Growth Rate | | | | | |
| Between Groups | 3.454e-005 | 2 | 1.727e-005 | F (2,10) = 0.09568 | P=0.9096 |
| Within Groups | 0.001805 | 10 | 0.0001805 |  |  |
| Total | 0.001840 | 12 |  |  |  |
