# Supplementary material for: Brain size reductions associated with endothelin B receptor mutation, a cause of Hirschsprung’s disease
Source: BMC Neurosci. 2021 Jun 19;22:42. doi: 10.1186/s12868-021-00646-z (PMC8214790; doi:10.1186/s12868-021-00646-z)
Supplement: Supplementary file 2 — Additional file 2: Table S2: One-way ANOVA showed statistically significant variations in the TBr, TCC, and Pit volumetric means of the studied rats: ETB+/+, ETB+/−, and ETB−/−. On the contrary, no significant variation was detected in other organ measurements of the three genotypes, as shown by A Post-hoc Tukey showed significant volumetric difference between the heterozygote (ETB+/−) and sl/sl (ETB−/−) in TBr (mean difference 113.7, SE 38.02, p = 0.0412), TCC (mean difference 52.07, SE 17.24, p = 0.0394), and Pit (mean difference 0.6113, SE 0.1927, p = 0.0315). However, measurement of wild type (ETB+/+) was not significantly different from those of the other two groups, as shown by B. [file 12868_2021_646_MOESM2_ESM.docx]

| Supplementary Table 2A : One-Way ANOVA for Volumetric Measurements | | | | | |
| --- | --- | --- | --- | --- | --- |
|  | Sum of Squares | df | Mean Square | F | Significance |
| TB Volume | | | | | |
| Between Groups | 25476 | 2 | 12738 | F (2,8) = 4.699 | P=0.0447 |
| Within Groups | 21685 | 8 | 2711 |  |  |
| Total | 47160 | 10 |  |  |  |
| TCC Volume | | | | | |
| Between Groups | 5252 | 2 | 2626 | F (2,8) = 4.711 | P=0.0445 |
| Within Groups | 4460 | 8 | 557.4 |  |  |
| Total | 9711 | 10 |  |  |  |
| TCP Volume | | | | | |
| Between Groups | 146.5 | 2 | 73.27 | F (2,8) = 3.072 | P=0.1023 |
| Within Groups | 190.8 | 8 | 23.85 |  |  |
| Total | 337.3 | 10 |  |  |  |
| OB Volume | | | | | |
| Between Groups | 27.66 | 2 | 13.83 | F (2,8) = 4.273 | P=0.0546 |
| Within Groups | 25.90 | 8 | 3.237 |  |  |
| Total | 53.56 | 10 |  |  |  |
| Med Volume | | | | | |
| Between Groups | 181.9 | 2 | 90.96 | F (2,8) = 1.394 | P=0.3023 |
| Within Groups | 521.9 | 8 | 65.23 |  |  |
| Total | 703.8 | 10 |  |  |  |
| Cer Volume | | | | | |
| Between Groups | 23.92 | 2 | 11.96 | F (2,8) = 0.8828 | P=0.4504 |
| Within Groups | 108.4 | 8 | 13.55 |  |  |
| Total | 132.3 | 10 |  |  |  |
| Pit Volume | | | | | |
| Between Groups | 0.7341 | 2 | 0.3671 | F (2,8) = 5.274 | P=0.0346 |
| Within Groups | 0.5568 | 8 | 0.0696 |  |  |
| Total | 1.291 | 10 |  |  |  |
| S&I Col Volume | | | | | |
| Between Groups | 4.240 | 2 | 2.120 | F (2,8) = 1.605 | P=0.2594 |
| Within Groups | 10.57 | 8 | 1.321 |  |  |
| Total | 14.81 | 10 |  |  |  |

| Supplementary Table 2B : Tukey post-hoc for Volumetric Measurements | | | | | | |
| --- | --- | --- | --- | --- | --- | --- |
| TB Volume | | | | | | |
| (A) | **(B)** | **Mean**  **Difference (A-B)** | **Std Error of Difference** | **Sig** | **Lower Bound** | **Upper Bound** |
| ET_B_^+/+^ | **ET_B_^+/-^** | -47.17 | 42.51 | 0.5350 | -168.6 | 74.30 |
| ET_B_^+/+^ | **ET_B_^-/-^** | 66.51 | 38.02 | 0.2463 | -42.13 | 175.2 |
| ET_B_^+/-^ | **ET_B_^-/-^** | 113.7 | 38.02 | 0.0412 | 5.036 | 222.3 |
| T-CC Volume | | | | | | |
| (A) | **(B)** | **Mean**  **Difference (A-B)** | **Std Error of Difference** | **Sig** | **Lower Bound** | **Upper Bound** |
| ET_B_^+/+^ | **ET_B_^+/-^** | -23.77 | 19.28 | 0.4683 | -78.85 | 31.32 |
| ET_B_^+/+^ | **ET_B_^-/-^** | 28.30 | 17.24 | 0.2843 | -20.97 | 77.57 |
| ET_B_^+/-^ | **ET_B_^-/-^** | 52.07 | 17.24 | 0.0394 | 2.801 | 101.3 |
| Pit Volume | | | | | | |
| (A) | **(B)** | **Mean**  **Difference (A-B)** | **Std Error of Difference** | **Sig** | **Lower Bound** | **Upper Bound** |
| ET_B_^+/+^ | **ET_B_^+/-^** | -0.2582 | 0.2154 | 0.4865 | -0.8737 | 0.3573 |
| ET_B_^+/+^ | **ET_B_^-/-^** | 0.3531 | 0.1927 | 0.2199 | -0.1974 | 0.9036 |
| ET_B_^+/-^ | **ET_B_^-/-^** | 0.6113 | 0.1927 | 0.0315 | 0.06078 | 1.162 |
