# Supplementary material for: Brain size reductions associated with endothelin B receptor mutation, a cause of Hirschsprung’s disease
Source: BMC Neurosci. 2021 Jun 19;22:42. doi: 10.1186/s12868-021-00646-z (PMC8214790; doi:10.1186/s12868-021-00646-z)
Supplement: Supplementary file 3 — Additional file 3: Table S3: One-way ANOVA comparison showed no significant difference was detected in the mean organ growth (A), volumetric/bodyweight ratios (B), and organ/whole-brain ratios (C) of the three genotype rats: ETB+/+, ETB+/−, and ETB−/−. These findings suggested that effect of ETB mutation was likely non-linear to gene doses. [file 12868_2021_646_MOESM3_ESM.docx]

| Supplementary Table 3A : One-Way ANOVA for Growth Measurements | | | | | |
| --- | --- | --- | --- | --- | --- |
|  | Sum of Squares | df | Mean Square | F | Significance |
| TB Growth | | | | | |
| Between Groups | 0.4669 | 2 | 0.2335 | F (2,8) = 2.162 | P=0.1776 |
| Within Groups | 0.8640 | 8 | 0.1080 |  |  |
| Total | 1.331 | 10 |  |  |  |
| TCC Growth | | | | | |
| Between Groups | 0.1620 | 2 | 0.08101 | F (2,8) = 3.692 | P=0.0731 |
| Within Groups | 0.1756 | 8 | 0.02195 |  |  |
| Total | 0.3376 | 10 |  |  |  |
| TCP Growth | | | | | |
| Between Groups | 0.004326 | 2 | 0.002163 | F (2,8) = 2.321 | P=0.1604 |
| Within Groups | 0.007456 | 8 | 0.0009320 |  |  |
| Total | 0.01178 | 10 |  |  |  |
| OB Growth | | | | | |
| Between Groups | 0.0003987 | 2 | 0.0001993 | F (2,8) = 2.426 | P=0.1502 |
| Within Groups | 0.0006574 | 8 | 8.217e-005 |  |  |
| Total | 0.001056 | 10 |  |  |  |
| Med Growth | | | | | |
| Between Groups | 0.004125 | 2 | 0.002062 | F (2,8) = 0.5419 | P=0.6015 |
| Within Groups | 0.03044 | 8 | 0.003805 |  |  |
| Total | 0.03457 | 10 |  |  |  |
| Cer Growth | | | | | |
| Between Groups | 5.588e-005 | 2 | 2.794e-005 | F (2,8) = 0.02401 | P=0.9763 |
| Within Groups | 0.009309 | 8 | 0.001164 |  |  |
| Total | 0.009365 | 10 |  |  |  |
| Pit Growth | | | | | |
| Between Groups | 2.830e-005 | 2 | 1.415e-005 | F (2,8) = 2.435 | P=0.1493 |
| Within Groups | 4.649e-005 | 8 | 5.811e-006 |  |  |
| Total | 7.479e-005 | 10 |  |  |  |
| S&I Col Growth | | | | | |
| Between Groups | 8.009e-005 | 2 | 4.005e-005 | F (2,8) = 0.2403 | P=0.8193 |
| Within Groups | 0.001568 | 8 | 0.0001960 |  |  |
| Total | 0.001648 | 10 |  |  |  |

| Supplementary Table 3B : One-Way ANOVA for Volumetric / Bodyweight ratios | | | | | |
| --- | --- | --- | --- | --- | --- |
|  | Sum of Squares | df | Mean Square | F | Significance |
| TB Volume / Bodyweight ratio | | | | | |
| Between Groups | 5.046 | 2 | 2.523 | F (2,8) = 0.2800 | P=0.7629 |
| Within Groups | 72.09 | 8 | 9.011 |  |  |
| Total | 77.14 | 10 |  |  |  |
| TCC Volume / Bodyweight ratio | | | | | |
| Between Groups | 3.427 | 2 | 1.713 | F (2,8) = 0.7730 | P=0.4932 |
| Within Groups | 17.73 | 8 | 2.216 |  |  |
| Total | 21.16 | 10 |  |  |  |
| TCP Volume / Bodyweight ratio | | | | | |
| Between Groups | 0.07151 | 2 | 0.03575 | F (2,8) = 3.3791 | P=0.6961 |
| Within Groups | 0.7544 | 8 | 0.09430 |  |  |
| Total | 0.8259 | 10 |  |  |  |
| OB Volume / Bodyweight ratio | | | | | |
| Between Groups | 0.001785 | 2 | 0.0008926 | F (2,8) = 0.04974 | P=0.9518 |
| Within Groups | 0.1436 | 8 | 0.01795 |  |  |
| Total | 0.1454 | 10 |  |  |  |
| Med Volume / Bodyweight ratio | | | | | |
| Between Groups | 0.03546 | 2 | 0.01773 | F (2,8) = 0.05759 | P=0.9444 |
| Within Groups | 2.463 | 8 | 0.3078 |  |  |
| Total | 2.498 | 10 |  |  |  |
| Cer Volume / Bodyweight ratio | | | | | |
| Between Groups | 0.008488 | 2 | 0.004244 | F (2,8) = 0.04889 | P=0.9526 |
| Within Groups | 0.6944 | 8 | 0.08680 |  |  |
| Total | 0.7029 | 10 |  |  |  |
| Pit Volume / Bodyweight ratio | | | | | |
| Between Groups | 0.0008387 | 2 | 0.0004193 | F (2,8) = 2.453 | P=0.1477 |
| Within Groups | 0.001368 | 8 | 0.0001710 |  |  |
| Total | 0.002207 | 10 |  |  |  |
| S&I Col Volume / Bodyweight ratio | | | | | |
| Between Groups | 0.01715 | 2 | 0.008575 | F (2,8) = 0.8153 | P=0.4762 |
| Within Groups | 0.08414 | 8 | 0.01052 |  |  |
| Total | 0.1013 | 10 |  |  |  |

| Supplementary Table 3C : One-Way ANOVA for Organ / Total brain ratios | | | | | |
| --- | --- | --- | --- | --- | --- |
|  | Sum of Squares | df | Mean Square | F | Significance |
| TCC / Total brain ratio | | | | | |
| Between Groups | 0.0007006 | 2 | 0.0003503 | F (2,8) = 1.699 | P=0.2427 |
| Within Groups | 0.001650 | 8 | 0.0002062 |  |  |
| Total | 0.002350 | 10 |  |  |  |
| TCP / Total brain ratio | | | | | |
| Between Groups | 1.830e-005 | 2 | 9.151e-006 | F (2,8) = 0.6731 | P=0.5368 |
| Within Groups | 0.0001088 | 8 | 1.360e-005 |  |  |
| Total | 0.0001271 | 10 |  |  |  |
| OB / Total brain ratio | | | | | |
| Between Groups | 6.605e-007 | 2 | 3.303e-007 | F (2,8) = 0.2201 | P=0.8071 |
| Within Groups | 1.200e-005 | 8 | 1.500e-006 |  |  |
| Total | 1.266e-005 | 10 |  |  |  |
| Med / Total brain ratio | | | | | |
| Between Groups | 2.227e-005 | 2 | 1.113e-005 | F (2,8) = 0.1216 | P=0.8871 |
| Within Groups | 0.0007325 | 8 | 9.157e-005 |  |  |
| Total | 0.0007548 | 10 |  |  |  |
| Cer / Total brain ratio | | | | | |
| Between Groups | 1.572e-005 | 2 | 7.859e-006 | F (2,8) = 0.2704 | P=0.7698 |
| Within Groups | 0.0002326 | 8 | 2.907e-005 |  |  |
| Total | 0.0002483 | 10 |  |  |  |
| Pit / Total brain ratio | | | | | |
| Between Groups | 2.332e-007 | 2 | 1.166e-007 | F (2,8) = 1.238 | P=0.3402 |
| Within Groups | 7.538e-007 | 8 | 9.423e-008 |  |  |
| Total | 9.871e-007 | 10 |  |  |  |
| S&I Col / Total brain ratio | | | | | |
| Between Groups | 2.379e-005 | 2 | 1.190e-005 | F (2,8) = 1.742 | P=0.2355 |
| Within Groups | 5.464e-005 | 8 | 6.830e-006 |  |  |
| Total | 7.843e-005 | 10 |  |  |  |
